# Mesenchymal stem cell transplantation for the infarcted heart: therapeutic potential for insulin resistance beyond the heart

**DOI:** 10.1186/1475-2840-12-128

**Published:** 2013-09-04

**Authors:** Curtis C Hughey, Lianli Ma, Freyja D James, Deanna P Bracy, Zhizhang Wang, David H Wasserman, Jeffrey N Rottman, Dustin S Hittel, Jane Shearer

**Affiliations:** 1 Department of Biochemistry and Molecular Biology, Faculty of Medicine, University of Calgary, 2500 University Drive N.W., Calgary, AB, Canada, T2N 1N4; 2 Mouse Metabolic Phenotyping Center, School of Medicine, Vanderbilt University, Nashville, TN 37240, USA; 3 Department of Molecular Physiology and Biophysics, School of Medicine, Vanderbilt University, Nashville, TN 37240, USA; 4 Department of Medicine, Division of Cardiovascular Medicine, School of Medicine, Vanderbilt University, Nashville, TN 37232, USA; 5 Faculty of Kinesiology, University of Calgary, Calgary, AB, Canada, T2N 1N4

**Keywords:** Diabetes, Glucose uptake, Isotopic tracer, Mitochondria, Myocardial infarction

## Abstract

**Background:**

This study aimed to evaluate the efficacy of mesenchymal stem cell (MSC) transplantation to mitigate abnormalities in cardiac-specific and systemic metabolism mediated by a combination of a myocardial infarction and diet-induced insulin resistance.

**Methods:**

C57BL/6 mice were high-fat fed for eight weeks prior to induction of a myocardial infarction via chronic ligation of the left anterior descending coronary artery. MSCs were administered directly after myocardial infarction induction through a single intramyocardial injection. Echocardiography was performed prior to the myocardial infarction as well as seven and 28 days post-myocardial infarction. Hyperinsulinemic-euglycemic clamps coupled with 2-[^14^C]deoxyglucose were employed 36 days post-myocardial infarction (13 weeks of high-fat feeding) to assess systemic insulin sensitivity and insulin-mediated, tissue-specific glucose uptake in the conscious, unrestrained mouse. High-resolution respirometry was utilized to evaluate cardiac mitochondrial function in saponin-permeabilized cardiac fibers.

**Results:**

MSC administration minimized the decline in ejection fraction following the myocardial infarction. The greater systolic function in MSC-treated mice was associated with increased *in vivo* cardiac glucose uptake and enhanced mitochondrial oxidative phosphorylation efficiency. MSC therapy promoted reductions in fasting arterial glucose and fatty acid concentrations. Additionally, glucose uptake in peripheral tissues including skeletal muscle and adipose tissue was elevated in MSC-treated mice. Enhanced glucose uptake in these tissues was associated with improved insulin signalling as assessed by Akt phosphorylation and prevention of a decline in GLUT4 often associated with high-fat feeding.

**Conclusions:**

These studies provide insight into the utility of MSC transplantation as a metabolic therapy that extends beyond the heart exerting beneficial systemic effects on insulin action.

## Background

The regional loss of cardiac myocytes following a myocardial infarction (MI) compromises the ability of the heart to pump blood to peripheral sites and may initiate compensatory mechanisms in an attempt to preserve cardiac function [1]. Researchers in regenerative medicine have become excited by the possibility of repairing and/or replacing pathological tissue via administration of exogenous cells [2-4]. Experimental study and clinical trials have identified cell-based therapies to consistently improve systolic function [5-10]. However, cell transplantation has been less than adequate at replacing the lost cells; which may be, in part, due to poor cell persistence following administration in the infarcted heart [11,12].

In the presence of poor cell survival and engraftment, the mechanisms promoting beneficial results reported following stem cell transplantation have been subject to an increasing number of studies that suggest cell-mediated paracrine effects are likely the major mediator for the improvement in cardiac function and the attenuation or slowing of the maladaptive processes [10]. The release of paracrine factors have a potential regulatory role in various processes that influence cardiac function including anti-apoptotic signalling and neovascularization as well as the modulation of inflammation, fibrosis, cardiac contractility, host stem cell activation and metabolism [10,13].

In terms of cardiac metabolism, animal studies indicate the ability of stem cell transplantation, specifically mesenchymal stem cell (MSC) transplantation, to lessen aberrations in glucose utilization, mitochondrial function and high-energy phosphate provision in the infarcted heart [14-18]. Since diabetes and heart disease often co-aggregate [19-21], the absence of metabolic dysfunction associated with type 2 diabetes in previous studies may not provide a complete model in assessing the utility of cell transplantation for the infarcted heart. Of particular interest is insulin resistance, as it enhances the risk of experiencing cardiovascular disease and a MI [22,23]. Moreover, individuals with insulin resistance and type 2 diabetes exhibit a greater probability of developing heart failure and higher mortality following a MI [22,24,25].

From a mechanistic perspective, insulin resistance may enhance cardiac pathology following a MI by inhibiting changes in metabolic characteristics that are initially suggested to be adaptive [26-30]. The cardiac metabolic phenotype shifts away from its predominant reliance on fatty acid oxidation towards increased glucose utilization early post-MI [26-30]. Glucose oxidation is more oxygen efficient and may provide a more effective means of ATP provision [31]. An alternative means of providing energy for cardiac contraction in an economical fashion involves the down-regulation of mitochondrial uncoupling proteins (UCPs) in the failing heart [31,32]. UCPs create an environment where oxygen consumption does not contribute to ATP synthesis, which is achieved through the uncoupling of the electrochemical gradient and ADP phosphorylation [33]. These adaptations in cardiac metabolic pathways may assist in meeting the energetic demand of contraction. However, this metabolic flexibility demonstrated by the heart is impeded in insulin resistance [34]. Insulin resistance inhibits glucose uptake creating a situation of severe detriment given both fatty acid and glucose metabolism are impaired and energy provision is compromised in the insulin resistant, infarcted heart [34]. Further insult occurs from hyperglycemia and hyperlipidemia [35,36]. For example, elevated circulating fatty acids promote an increase in cardiac UCP3 levels [37]. The increase in UCP3 may stimulate higher mitochondrial uncoupling and further energy demand/supply disparities.

Given the deleterious impact of insulin resistance on the infarcted heart, this study aimed to identify whether the efficacy of MSC administration to minimize alterations in metabolic processes that assist in ATP provision following a MI is maintained in a murine model of diet-induced insulin resistance/MI.

## Methods

**Figure 1:**
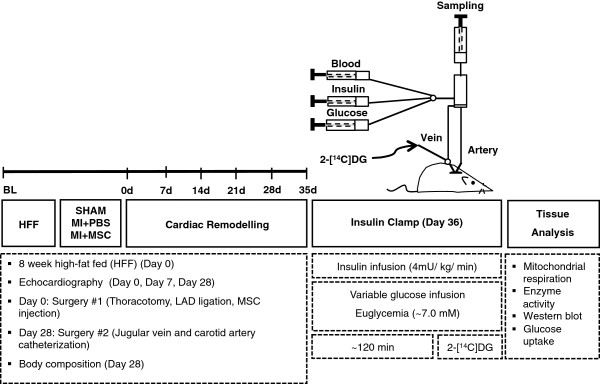
**Schematic representation of experimental procedures and timeline.** Echocardiography on conscious mice was performed prior to, seven and 28 days following ligation of the left anterior descending coronary artery (LAD). Arterial and jugular catheterization was performed 28 days following the LAD ligation for the sampling and infusion protocols of the hyperinsulinemic-euglycemic (insulin) clamp. Insulin clamps were performed following seven days of recovery from the catheterization surgeries (36 days post-LAD ligation/13 weeks of high-fat feeding) to assess insulin action in the conscious, unrestrained mouse. Isotopic tracer 2-[^14^C]deoxyglucose (2-[^14^C]DG) administration during the insulin clamps allowed for whole body and tissue-specific substrate uptake to be assessed *in vivo*. Additional experiments included evaluation of mitochondrial respiration in permeabilized cardiac tissue and key molecular regulators of metabolism by immunoblotting. MI + PBS, myocardial infarction + phospho-buffered saline; MI + MSC, myocardial infarction + mesenchymal stem cells.

See Figure 1 for a schematic of experimental outline.

### Animal characteristics and myocardial infarction

Procedures were approved by the Vanderbilt University Animal Care and Use Committee and performed according to the Guide for the Care and Use of Laboratory Animals published by the National Institutes of Health (NIH Publication No. 85–23, revised 1996, A3227-01). Thirteen week-old, eight week high-fat fed (HFF) (F3282, 60% calories from fat, Bio-Serv, Frenchtown, NJ, USA), C57BL/6J, male mice (Jackson Laboratories, Bar Harbour, ME, USA) were randomly separated into three groups: SHAM, MI + phosphate buffered saline (MI + PBS) and MI + mesenchymal stem cells (MI + MSC). A left anterior descending coronary artery (LAD) chronic ligation induced a MI as described [18]. Following the ligation, 25 μl of PBS (pH 7.2) or 2.5 × 10^5^ MSCs in 25 μl of PBS (pH 7.2) were injected into the blanching peri-infarct area of the MI + PBS or MI + MSC mice, respectively.

### Mesenchymal stem cells

MSCs reputedly display immunoprivileged characteristics that make the use of this cell type attractive for cell transplantation purposes [38-40]. These unique characteristics provide promise for use of MSCs in not only autologous transplantation but allogenic and xenogenic transplantation. Given this, human MSCs were purchased from the Texas A&M Health Science Center College of Medicine Institute for Regenerative (Temple, TX, USA) that provides standardized MSC preparations through support from the NIH/NCRR (P40 RR 17447–06). The MSCs have been described to consistently exhibit trilineage potential and be CD90^+^, CD105^+^, CD49c^+^, CD49f^+^, CD166^+^, CD105^+^, CD29^+^, CD44^+^, CD45^-^, CD34^-^, CD117^-^ and CD36^-^[41]. MSCs were expanded in alpha minimum essential medium (αMEM) with L-glutamine (Life Technologies, Burlington, ON, Canada), 16.5% defined FBS (HyClone, Logan, UT, USA), penicillin (100 units/ml) and streptomycin (100 μg/ml). All mice received MSCs from a single donor and passages 3–8 were utilized.

### Cardiac performance

To assess cardiac contractile function, M-mode echocardiography was completed in conscious mice at baseline, seven and 28 days post-MI as previously described [42,43].

### Catheterization procedures

Chronic catheterization surgeries were performed 28 days post-ligation (12 weeks HFF) as those previously described for hyperinsulinemic-euglycemic (insulin clamps) [18]. Post-surgery, the mice were housed individually for seven days to ensure the mice were within 10% of pre-surgical weight prior to insulin clamps.

### Hyperinsulinemic-euglycemic clamps

Our laboratory has shown that insulin resistance is induced in 12 weeks HFF C57BL/6 mice [44,45]. Insulin clamps were performed with procedural considerations previously described [46] at 36 days post-MI (13 weeks HFF). Mice were fasted at 7:00 a.m., five hours prior to initiation of the insulin clamps. One hour prior to the experiment, the externalized mouse catheters were connected to catheter extensions attached to infusion syringes. Just prior to the onset of the insulin clamp, an arterial blood sample was obtained to evaluate levels of arterial glucose, insulin, non-esterified fatty acids (NEFA) and hematocrit. Each experiment consisted of a continuous infusion (4 mU/kg/min) of insulin. Euglycemia (~7.0 mM) was maintained during the insulin clamps. Venous infusion of saline-washed erythrocytes (5 μl/min) during the insulin clamp prevented a decline in hematocrit due to arterial sampling. Insulin clamp duration averaged 122.07 ± 9.48 minutes until a stable glucose infusion rate (GIR) and euglycemia was achieved for at least 30 minutes. Arterial blood was sampled to determine glucose levels (t = 0 minutes) had achieved the steady state. Immediately following the blood sampling, a bolus containing 2-[^14^C]deoxyglucose (2-[^14^C]DG; 481 kBq) was administered into the jugular vein to provide an index of tissue-specific glucose uptake. At *t* = 2, 5, 10, 15, 20 minutes, arterial blood was sampled to determine glucose and 2-[^14^C]DG. At *t* = 30 minutes, arterial blood was taken for the measurement of glucose, experimental insulin, 2-[^14^C]DG and hematocrit. Plasma was stored at −20°C until analysis. Following the insulin clamp, mice were killed via cervical dislocation and tissues were immediately excised for analysis or stored at −80°C. The heart, soleus, gastrocnemius, superficial vastus lateralis and white adipose tissue from the epididymal fat pad were collected.

### Plasma analyses

Arterial insulin was assayed via a double antibody method [47]. Plasma NEFAs (NEFA C kit; Wako Chemicals, Richmond, VA, USA) and glucose were determined spectrophotometrically as previously described [18]. Plasma 2-[^14^C]DG was assessed as previously outlined [45].

### Tissue-specific substrate kinetics

Tissue 2-[^14^C]DG and tissue phosphorylated 2-[^14^C]DG (2-[^14^C]DG-P) were determined as previously described [45]. The metabolic index of glucose (R_*g*_) uptake was calculated [48] and expressed as previously described [49].

### Mitochondrial respiration and enzymatic measurements

Cardiac peri-infarct fibers were prepared and saponin-permeabilized as described [50]. High-resolution respirometry (Oroboros Instruments, Innsbruck, Austria) was performed in duplicate at 37°C in MiR05 (Final concentration: 0.5 mM EGTA, 3 mM MgCl_2_·6H_2_O, 20 mM taurine, 10 mM KH_2_PO_4_, 20 mM HEPES, 1 g/L BSA, 60 mM potassium-lactobionate, 110 mM sucrose, pH 7.1, adjusted at 30°C). Substrates (final concentration) included 10 mM glutamate plus 2 mM malate and 5 mM pyruvate, 5 mM ADP and 10 mM succinate. 10 mM cytochrome c was added to ensure the outer mitochondrial membrane was intact after processing. Peri-infarct citrate synthase activity was determined via previous methods [18].

### Immunoblotting

Tissues were homogenized in a lysis buffer (final concentration) containing 20 mM NaCl, 20 mM Tris–HCl, 0.1 mM EDTA, 1% Triton X-100, 0.5% (wt./vol.) sodium deoxycholate, and 0.1% β-Mercapto Ethanol (vol./vol.) (pH 7.4) in the presence of a protease inhibitor cocktail (Sigma-Aldrich, Oakville, ON, Canada) and phosphatase inhibitor cocktail (Thermo Fisher Scientific, Mississauga, ON, Canada). Cardiac (20–30 μg), gastrocnemius (40 μg) and white adipose tissue (40 μg) proteins were resolved on NuPAGE 4-12% (vol./vol.) Bis-Tris gels (Life Technologies) and transferred to a polyvinylidene fluoride membrane. Membranes were probed with peroxisome proliferator-activated receptor gamma coactivator-1alpha (PGC-1α; Santa Cruz Biotechnology, Santa Cruz, CA, USA), glucose transporter 4 (GLUT4; Abcam, Cambridge, MA, USA), hexokinase II (HKII; Chemicon, Temecula, CA, USA), UCP3 (Abcam), phospho-Akt(Ser473) (p-Akt; Cell Signaling Technology, Whitby, ON, Canada) and Akt (Cell Signaling Technology) and oxidative phosphorylation complexes I-V (OXPHOS CI-CV; Abcam) antibodies. A goat-anti-mouse secondary antibody (Thermo Fischer Scientific) was used for the OXPHOS CI-CV primary antibody cocktail. A goat-anti rabbit secondary antibody (Cell Signaling Technologies) was used for all other primary antibodies. Glyceraldehyde 3-phosphate dehydrogenase (GAPDH; Abcam) expression was employed as a control for all immunoblots.

### Statistical analyses

ANOVA or two-way repeated measures ANOVA were performed to detect statistical differences (p < 0.05) as appropriate followed by Tukey’s post hoc tests. All data are reported as means ± SEM.

## Results

### Stem cell therapy promotes improvement in cardiac contractile function

**Figure 2:**
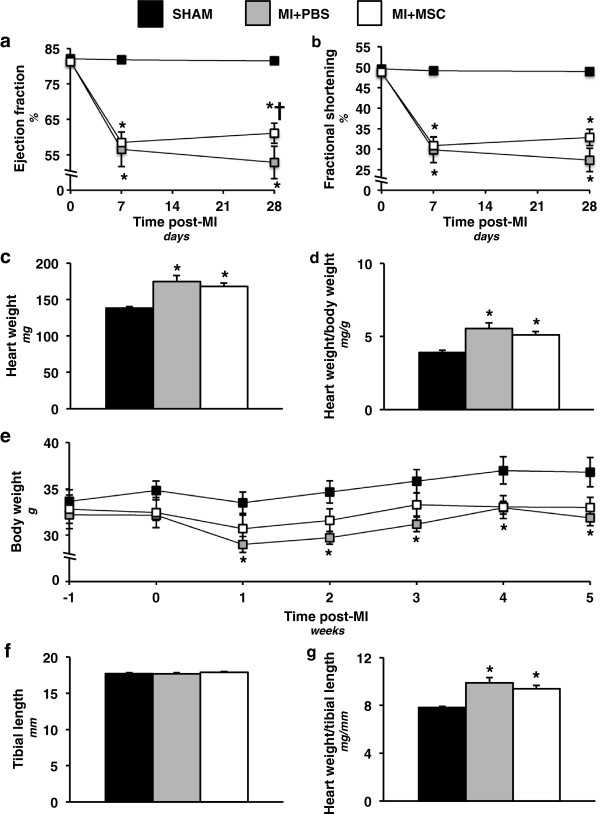
**Cardiac functional and hypertrophic indices. (a)** Cardiac ejection fraction (%) prior to, seven and 28 days following a MI. n = 8-13 mice per group. **(b)** Cardiac fractional shortening (%) prior to, seven and 28 days following a MI. n = 8-13 mice per group. **(c)** Heart weight 36 days post-MI surgery. n = 11-14 mice per group. **(d)** Heart weight-to-body weight ratio 36 days following a MI. n = 11-14 mice per group. **(e)** Time course of body weight from one week prior to chronic left anterior descending coronary artery ligation to five weeks post-ligation. n = 10-12 mice per group. **(f)** Tibial length 36 days following a MI. n = 11-14 mice per group. **(g)** Heart weight-to-tibial length 36 days post-MI. n = 11-14 mice per group. Data are mean ± S.E.M. *p < 0.05 vs. SHAM. †p < 0.05 vs. MI + PBS.

Contractile abnormalities were observed using echocardiography (Table 1) in the MI + PBS group seven and 28 days following a MI as indicated by the depression in ejection fraction (Figure 2a) and fractional shortening (Figure 2b). The MSC-treated animals displayed a comparable reduction in ejection fraction as the MI + PBS group at seven days post-MI (Figure 2a). However, the MSC-treated hearts exhibited a greater ejection fraction than the MI + PBS mice at 28 days following cardiac insult (Figure 2a). The MI + MSC mice, similar to the MI + PBS animals, displayed a depression in fractional shortening at seven and 28 days post-MI (Figure 2b).

**Table 1:** Cardiovascular parameters in conscious, high-fat fed, C57BL/6 mice

	**Baseline**			**7 days post-MI**			**28 days post-MI**		
	**SHAM**	**MI + PBS**	**MI + MSC**	**SHAM**	**MI + PBS**	**MI + MSC**	**SHAM**	**MI + PBS**	**MI + MSC**
**HR (bpm)**	647 ± 16	662 ± 16	689 ± 9*	720 ± 8	699 ± 10	713 ± 12	710 ± 11	720 ± 13	731 ± 6
**FS (%)**	49.58 ± 0.75	48.88 ± 1.02	48.74 ± 0.46	49.12 ± 0.78	29.88 ± 3.16*	30.90 ± 2.03*	48.99 ± 0.70	27.34 ± 2.84*	32.90 ± 2.02*
**EF (%)**	82.07 ± 0.72	81.37 ± 1.01	81.23 ± 0.45	81.80 ± 0.76	56.51 ± 4.88*	58.48 ± 2.9*	81.48 ± 0.74	52.80 ± 2.93*	61.10 ± 2.88*†
**IVSd (mm)**	0.77 ± 0.02	0.81 ± 0.02	0.79 ± 0.01	0.76 ± 0.02	0.73 ± 0.04	0.84 ± 0.01*†	0.79 ± 0.01	0.76 ± 0.03	0.85 ± 0.01*†
**LVIDd (mm)**	3.19 ± 0.07	3.21 ± 0.08	3.28 ± 0.04	3.02 ± 0.04	4.31 ± 0.24*	4.01 ± 0.13*	3.23 ± 0.08	4.32 ± 0.21*	4.27 ± 0.13*
**LVPWd (mm)**	0.72 ± 0.01	0.69 ± 0.02	0.80 ± 0.02†	0.74 ± 0.02	0.68 ± 0.07	0.86 ± 0.03*†	0.77 ± 0.02	0.71 ± 0.04	0.91 ± 0.03
**IVSs (mm)**	0.91 ± 0.02	0.91 ± 0.02	0.90 ± 0.01	0.88 ± 0.02	0.87 ± 0.05	0.92 ± 0.02	0.88 ± 0.01	0.84 ± 0.02	0.96 ± 0.01*†
**LVIDs (mm)**	1.61 ± 0.04	1.65 ± 0.06	1.68 ± 0.03	1.54 ± 0.04	3.06 ± 0.28*	2.79 ± 0.16*	1.65 ± 0.06	3.17 ± 0.26*	2.89 ± 0.17*
**LVPWs (mm)**	0.97 ± 0.02	0.98 ± 0.04	1.04 ± 0.01	0.88 ± 0.02	0.83 ± 0.09	1.01 ± 0.02*†	0.96 ± 0.02	0.82 ± 0.06*	1.11 ± 0.03*†

Data are mean ± SEM for n = 8-13 mice per group.

*p < 0.05 vs. SHAM at specified time point.

†p < 0.05 vs. MI + PBS at specified time point.

*Abbreviations:* HR, heart rate; FS, fractional shortening; EF, ejection fraction; IVSd, interventricular septal thickness in diastole; LVIDd, left ventricle (LV) end-diastolic dimension; LVPWd, LV posterior wall thickness in diastole; IVSs, interventricular septal thickness in systole; LVIDs, LV end-systolic dimension; LVPWs, LV posterior wall thickness in systole.

Indices of cardiac structural alterations were also assessed. Heart weight (Figure 2c) and heart-to-body weight (Figure 2d) were increased in MI + PBS mice 36 days post-MI. A comparable elevation in these hypertrophy markers was observed in the MSC-treated mice (Figure 2c,d). Of note, measurement of body weight from one week prior to the ligation surgery to five weeks post-MI indicated that the MI + PBS mice were lower as a result of the MI (Figure 2e). In contrast, mice receiving MSC transplantation displayed a comparable body weight to animals of the SHAM group (Figure 2e). Given the changes in body weight, tibial length was used to normalize heart weight and provide another index of pathological cardiac hypertrophy. Tibial length was unchanged between groups (Figure 2f). Both infarcted groups displayed an elevated heart weight-to-tibial length compared to the SHAM group (Figure 2f) indicating a hypertrophic response to the infarct that was not inhibited by the MSC therapy.

### Enhanced insulin-stimulated cardiac glucose utilization in MSC-treated mice

**Figure 3:**
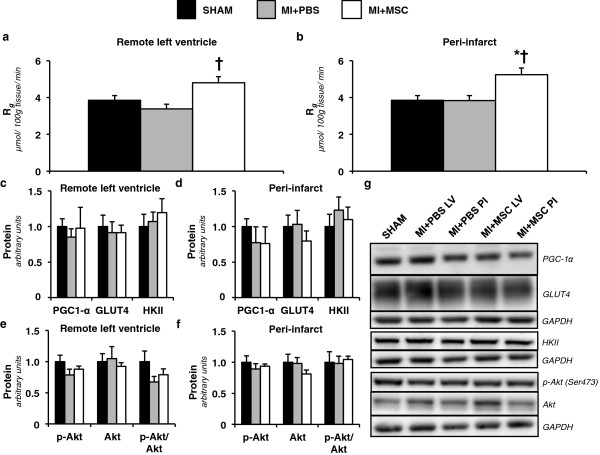
**Regional insulin-stimulated cardiac glucose uptake. (a)** Metabolic index of glucose uptake (*R*_*g*_) in the remote left ventricle and **(b)** peri-infarct region of the left ventricle. Cardiac R_*g*_ values are relative to brain R_*g*_. n = 8-9 mice per group. **(c)** Remote left ventricle and **(d)** peri-infarct peroxisome proliferator-activated receptor gamma coactivator-1alpha (PGC-1α), glucose transporter 4 (GLUT4) and hexokinase II (HKII) as determined by immunoblotting. **(e)** Left ventricle and **(f)** peri-infarct phospho-Akt (p-Akt), Akt and p-Akt-to-total Akt ratio (p-Akt/Akt) as determined by immunoblotting. **(g)** Representative immunoblotting performed to measure PGC-1α, GLUT4, HKII, p-Akt and Akt. Cardiac proteins are normalized to glyceraldehyde-3-phosphate dehydrogenase (GAPDH) content and are relative to the SHAM group. n = 6 mice per group. Data are mean ± S.E.M. *p < 0.05 vs. SHAM. †p < 0.05 vs. MI + PBS.

Isotopic glucose **(**2-[^14^C]DG) was administered during the insulin clamps to assess tissue-specific glucose uptake. A MI did not influence regional insulin-stimulated glucose uptake (R_*g*_) in the heart. The remote left ventricle (MI + PBS LV) and the peri-infarct region (MI + PBS PI) in the MI + PBS animals were comparable to the SHAM group (Figure 3a,b). In contrast, intramyocardial injection of MSCs enhanced cardiac R_*g*_ in response to insulin. The remote left ventricle of the MSC-treated mice (MI + MSC LV) displayed greater R_*g*_ compared to that of the MI + PBS mice (Figure 3a). Furthermore, the insulin-mediated response of the peri-infarct region of the MI + MSC hearts (MI + MSC PI) was more apparent. The MI + MSC PI region exhibited a higher R_*g*_ than the SHAM hearts and the peri-infarct region of the MI + PBS group (Figure 3b).

To identify the mechanisms by which MSC administration promoted cardiac glucose uptake key regulators of glucose metabolism were evaluated. The remote left ventricle and peri-infarct PGC-1α, GLUT4 and HKII were comparable between groups (Figure 3c,d). The insulin signalling pathway was also probed by determining p-Akt(Ser473) and total Akt. Again, the regional p-Akt-to-Akt ratio was similar between groups (Figure 3e,f).

### MSC transplantation augments mitochondrial physiology

**Figure 4:**
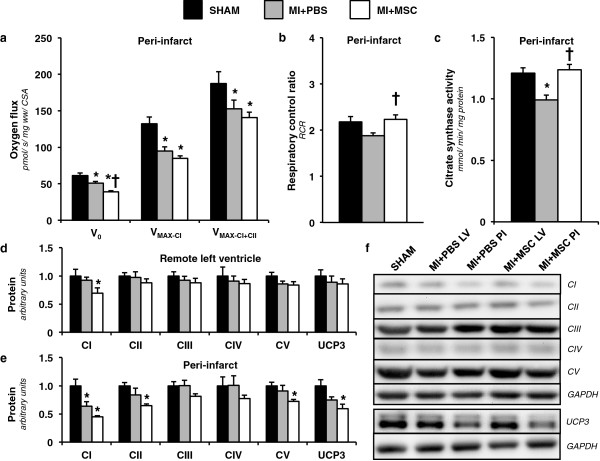
**Cardiac mitochondrial function and characteristics. (a)** Peri-infarct permeabilized cardiac fiber basal oxygen consumption supported by glutamate, malate and pyruvate (V_0_), maximal oxygen consumption (ADP-stimulated) supported by glutamate, malate and pyruvate through complex I (V_MAX-CI_) and maximal convergent oxygen flux supported by glutamate, malate, pyruvate and succinate (V_MAX-CI+CII_). n = 8-9 mice per group. **(b)** Respiratory control ratio (RCR; defined as V_MAX-CI_/V_0_). n = 8-9 mice per group. **(c)** Peri-infarct citrate synthase activity (CSA) (mmol/min/mg protein). n = 8-9 mice per group. **(d)** Remote Left ventricle and **(e)** peri-infarct mitochondrial oxidative phosphorylation (OXPHOS) complexes I-V (CI-CV) and uncoupling protein 3 (UCP3) as determined by immunoblotting. **(f)** Representative immunoblotting of the regional protein levels OXPHOS complexes I-V and UCP3. Cardiac protein was normalized to glyceraldehyde-3-phosphate dehydrogenase (GAPDH) content and expressed relative to the SHAM group. n = 6 mice per group. Data are mean ± S.E.M. *p < 0.05 vs. SHAM. †p < 0.05 vs. MI + PBS.

Given both contractile function and substrate utilization are influenced by energy metabolism, polarographic oxygen flux measurements were performed to assess integrative mitochondrial OXPHOS. The MI + PBS peri-infarct fibers exhibited a reduced basal oxygen consumption (V_0_) supported by glutamate, malate and pyruvate compared to the SHAM group (Figure 4a). Interestingly, MSC-treated hearts displayed an even further depression in basal oxygen flux compared to the MI + PBS and SHAM peri-infarct regions (Figure 4a). ADP-stimulated oxygen consumption through complex I was reduced in MI + PBS and MI + MSC animals (V_MAX-CI_; Figure 4a). Similarly, ADP-stimulated oxygen flux via convergent electron flux through mitochondrial complexes I + II was reduced in both infarcted groups (V_MAX-CI + CII_; Figure 4a). Intriguingly, the respiratory control ratio (RCR) was reduced in the MI + PBS hearts compared with the MI + MSC cardiac fibers (Figure 4b). This suggests a decrease in OXPHOS efficiency in the MI + PBS mice. Citrate synthase activity was assessed to identify differences in mitochondrial content. The MI + PBS group exhibited a decline in citrate synthase activity (Figure 4c). The preservation of cardiac citrate synthase activity in the MI + MSC mice prompted the evaluation of mitochondrial proteins in an effort to identify mechanisms contributing to the reduced V_0_ but greater RCR. Protein expression of mitochondrial OXPHOS complexes showed the MI + MSC mice to have a lower complex I protein in the remote left ventricle region (Figure 4d,f). Similarly, the MI + PBS and MI + MSC peri-infarct region exhibit a reduced complex I protein compared to the SHAM heart (Figure 4e,f). Surprisingly, there was an added effect of the MSC therapy. The MI + MSC peri-infarct region displayed a lower complex II and complex V compared to the SHAM hearts (Figure 4e,f). Another potential contributor to the lower basal oxygen flux but elevated RCR is the peri-infarct UCP3 that was lower in the MI + MSC mice (Figure 4e,f).

### Reduced fasting plasma glucose and fatty acids following MSC administration

High-fat diets have been reported to promote an increase in tissue UCP3 protein. More specifically, cardiac UCP3 positively correlates with circulating fatty acid levels [37]. As such, we evaluated fasting plasma non-esterified fatty acid (NEFA) concentration. There was a lowering of NEFA levels in the MI + MSC mice (Table 2). In addition, fasting plasma glucose concentration was reduced following MSC therapy (Table 2). Of note, arterial insulin concentrations in the fasted state and during the insulin clamp were both similar between groups (Table 2).

**Table 2:** Biometric characteristics of high-fat fed, C57BL/6 mice

	**SHAM**	**MI + PBS**	**MI + MSC**
**Fasting Plasma Glucose (mM)**	11.37 ± 0.48	10.81 ± 0.43	9.06 ± 0.32*†
**Fasting Plasma NEFA (mM)**	1.04 ± 0.05	1.09 ± 0.06	0.8 ± 0.06*†
**Fasting Plasma Insulin (μU/ml)**	60.08 ± 13.22	50.76 ± 16.15	42.46 ± 11.13
**Experimental Plasma Insulin (μU/ml)**	128.02 ± 15.77	108.17 ± 8.84	160.04 ± 24.32
**Muscle (%)**	68.70 ± 1.76	77.75 ± 1.96*	67.88 ± 2.38†
**Fat (%)**	29.53 ± 1.80	20.74 ± 2.02*	30.25 ± 2.42†
**Free Fluid (%)**	1.77 ± 0.11	1.51 ± 0.17	1.87 ± 0.27

Data are mean ± SEM.

n = 11-12 mice per group for plasma glucose, n = 10-12 for plasma NEFA, n = 8-9 for fasting plasma insulin, n = 8 for experimental plasma insulin, n = 9-13 for body composition.

*p < 0.05 vs. SHAM.

†p < 0.05 vs. MI + PBS.

### Systemic insulin sensitivity elevated by MSC injection

**Figure 5:**
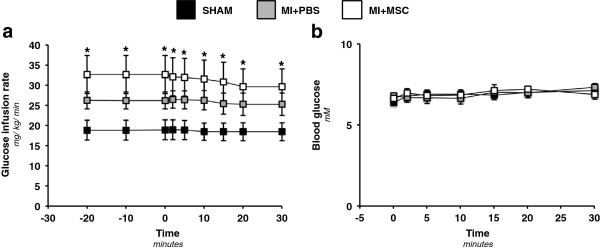
**Whole body insulin sensitivity and blood glucose during the hyperinsulinemic-euglycemic (insulin) clamp. (a)** Glucose infusion rate (GIR) during the insulin clamp. The GIR is equivalent to the whole body glucose disposal rate in response to venous insulin infusion. The insulin infusion was for approximately 120 minutes prior to 2-[^14^C]deoxyglucose (2-[^14^C]DG) administration. The GIR is displayed as a time course commencing twenty minutes prior to administration of 2-[^14^C]DG (−20 minute time point) to 30 minutes following 2-[^14^C]DG infusion (30 minute time point). **(b)** Arterial, blood glucose concentration following 2-[^14^C]DG for 30 minutes post-administration. Data are mean ± SEM for n = 9-12 mice per group. *p < 0.05 vs. SHAM.

The lowering of fasting plasma glucose and fatty acids suggested a systemic effect of the MSC administration. To this end, insulin clamps were performed in the conscious, unrestrained mouse to evaluate whole body glucose disposal in response to insulin *in vivo*. The GIR required to maintain an arterial glucose concentration of 7.0 mM was elevated in MI + MSC mice throughout the duration of the insulin clamp (Figure 5a). The GIR for the MI + PBS mice was similar to the SHAM group (Figure 5a). Arterial glucose was comparable between groups during the insulin clamp (Figure 5b).

### Increased peripheral tissue glucose uptake post-MSC therapy

**Figure 6:**
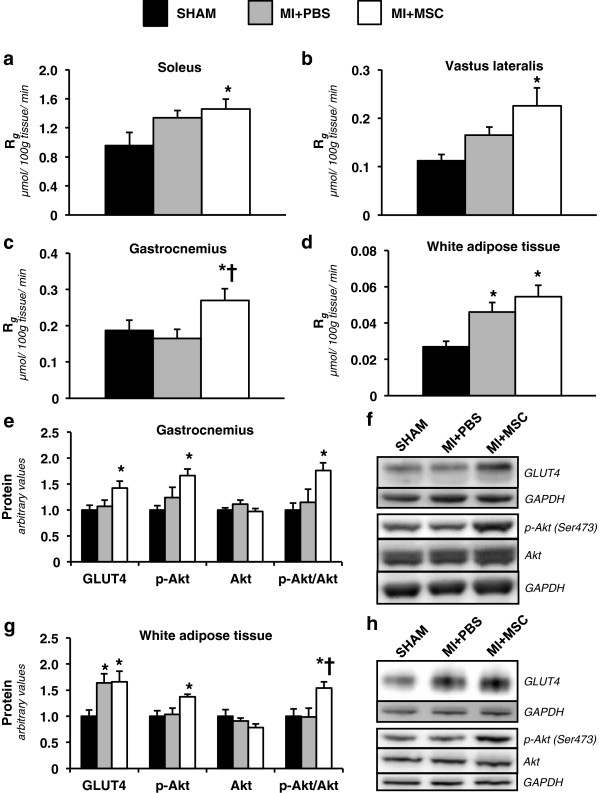
**Insulin-stimulated peripheral tissue glucose utilization. (a)** Metabolic index of glucose uptake (R_*g*_) in the soleus. **(b)** Metabolic index of glucose uptake (R_*g*_) in the superficial vastus lateralis. **(c)** Metabolic index of glucose uptake (R_*g*_) in the gastrocnemius. **(d)** Metabolic index of glucose uptake (R_*g*_) in white adipose tissue. Tissue R_*g*_ values are relative to brain R_*g*_. n = 9 mice per group. **(e)** Gastrocnemius glucose transporter 4 (GLUT4), phospho-Akt (p-Akt), Akt and p-Akt-to-total Akt ratio (p-Akt/Akt) as determined by immunoblotting. n = 6-8 mice per group. **(f)** Representative immunoblotting performed to measure gastrocnemius GLUT4, p-Akt and Akt. **(g)** White adipose tissue glucose transporter 4 (GLUT4), phospho-Akt (p-Akt), Akt and p-Akt-to-total Akt ratio (p-Akt/Akt) as determined by immunoblotting. n = 6 mice per group. **(h)** Representative immunoblotting performed to measure white adipose tissue GLUT4, p-Akt and Akt. Protein levels are normalized to glyceraldehyde-3-phosphate dehydrogenase (GAPDH) content and are expressed relative to the SHAM group. Data are mean ± S.E.M. *p < 0.05 vs. SHAM. †p < 0.05 vs. MI + PBS.

In agreement with the increased GIR, the MSC administration increased insulin-stimulated R_*g*_ in peripheral tissues. The MI + MSC soleus, superficial vastus lateralis, gastrocnemius and white adipose tissue had a greater R_*g*_ compared to the same tissues of the SHAM group (Figure 6a-d). The MI + MSC gastrocnemius also exhibited an elevated R_*g*_ compared to that of the MI + PBS mice (Figure 6c). The MI + PBS mice displayed a higher R_*g*_ in white adipose tissue compared to the SHAM group (Figure 6d). The protein expression of regulators involved in insulin-mediated R_*g*_ was evaluated in the gastrocnemius and white adipose tissue. GLUT4 was higher in the MI + MSC gastrocnemius compared to the SHAM group (Figure 6e,f). Also, gastrocnemius p-Akt and the p-Akt-to-Akt ratio were greater in the MI + MSC mice (Figure 6e,f). Both the MI + MSC and MI + PBS groups displayed higher GLUT4 in the white adipose tissue (Figure 6g,h). However, only the MI + MSC exhibited elevated p-Akt relative to the SHAM group in white adipose tissue (Figure 6g,h). Furthermore, the p-Akt-to-Akt ratio in MI + MSC white adipose tissue was greater than that of the SHAM and MI + PBS mice (Figure 6g).

## Discussion

Numerous studies have reported that cell-based therapeutics can minimize insult to the infarcted heart, in large part, through trophic mechanisms [10,13]. However, as stem cell therapy transfers to clinical trials and ideally to routine use in the clinical setting a clearer understanding as to how the transplanted cells improve outcomes is needed. To gain greater insight into the mechanisms of action, stem cell administration in models of physiological relevance is required. Few studies have included type 2 diabetes and/or its associated metabolic dysregulation on the effect of stem cell transplantation in the infarcted heart. Yan et al. [51] assessed the influence of induced-pluripotent stem (iPS) cell administration in the db/db mouse heart following a MI. Echocardiography revealed that 14 days post-MI left ventricle contractile dysfunction was minimized by iPS cell transplantation [51]. The current study expands on the work of Yan and colleagues [51] by employing a diet-induced insulin resistant C57BL/6 mouse model and assessing the ability of MSC administration to minimize cardiac systolic abnormalities post-infarct. We report the MSC therapy to improve cardiac ejection fraction 28 days post-MI. This indicates that MSC transplantation holds similar potential in restoring left ventricle systolic function in the presence of diet-induced systemic metabolic insults. Furthermore, our experiments investigate beyond cardiac contractile function to evaluate the effect of MSC transplantation on metabolic characteristics that may minimize dysfunction.

The superior contractile function in the MSC-treated mice may be due to the ability of the hearts to more effectively utilize substrates and synthesize ATP. The current study demonstrates intramyocardial administration of MSCs to improve the ability of the heart to utilize glucose in the remote left ventricle and peri-infarct region. This improvement in glucose uptake could assist in energy provision. To provide insight into the means through which the MSC transplantation elevated cardiac glucose uptake molecular regulators of glucose metabolism were evaluated. The current study did not expose alterations in regional cardiac levels of PGC-1α, GLUT4, HKII, p-Akt, Akt and the p-Akt-to-Akt ratio. This suggests key proteins involved in glucose uptake and insulin signalling were not augmented by the MSC treatment in the heart. The absence of changes in these proteins led us to explore mitochondrial function as a possible mechanism by which glucose would be utilized at a higher rate.

Downstream of glucose transport and glycolysis, mitochondria OXPHOS acts as a link between glucose uptake and ATP generation. Early studies evaluating the effect of MSC transplantation show an improvement in the peri-infarct and whole left ventricle phosphocreatine (PCr)-to-ATP ratio [14-16]. The PCr-to-ATP ratio reflects efficiency of myocardial energy provision, and correlates well with left ventricle contractile function [14,52-54]. Furthermore, MSC therapy reduces basal mitochondrial oxygen consumption (V_0_) and improves cardiac RCR (V_MAX-CI_/V_0_) [18]. V_0_ represents futile respiration that does not contribute to ATP synthesis and a lower basal oxygen flux helps increase RCR; which is indicative of a more efficient OXPHOS. Mitochondrial dysfunction has been theorized to promote insulin resistance [55]. Conversely, improved mitochondrial function may help alleviate impaired glucose uptake and assist in greater ATP synthesis. In the current study, MSC therapy was also able to lower V_0_ and increase RCR. The ability of MSCs to promote these alterations on mitochondrial function may be due to a direct influence on OXPHOS complexes. Jameel et al. [56] indicate that MSC administration for the infarcted heart reduces gene expression of complex I subunits. We report that in the peri-infarct region, there was a decline in complex I protein levels following a MI. However, MSC transplantation enhanced the reduction in complex I as indicated by the decline induced in the remote left ventricle and peri-infarct region. Complex II and V were also lower in the MSC-treated peri-infarct region. A reduction in OXPHOS complexes I, II and V may explain the diminished V_0_ as a repressed complex number would subdue electron flux and oxygen consumption. Another potential reason for the lower V_0_ and elevated RCR is alterations in UCP3. UCP3 has been suggested to function as a potential OXPHOS uncoupler [57]. As such, a reduction in protein may promote a decline in UCP3-mediated uncoupling.

Increases in tissue UCP3 are often more pronounced with high-fat diets and with elevated circulating fatty acids [37,58]. We report that MSC-treated mice exhibited a reduction in fasted plasma NEFAs. This decline would promote a lower tissue expression of UCP3. Further systemic influence of the MSC therapy was observed in fasting plasma glucose. We found plasma glucose levels were diminished in the MSC-treated animals. In agreement, animal studies indicate that MSC therapy is capable of lowering blood glucose levels in both type 1 diabetes [59,60] and type 2 diabetes [61]. A recent pilot clinical study has reported a reduction in blood glucose in individuals with type 2 diabetes following MSC transplantation [62].

The exact means by which MSC exert these effects on metabolism have yet to be elucidated. To date, the majority of studies evaluating stem cell transplantation in diabetes have focused on the ability of the administered cells to regenerate and/or protect the insulin-producing beta cells of the pancreas as a means of providing improved glucose control and reducing diabetic complications [63]. To the authors’ knowledge, only a single study has evaluated the influence of cell-based therapy on peripheral tissue glucose disposal as a mechanism through which the treatment reduces hyperglycemia. Si et al. [61] employed insulin clamps to test whether intravenous MSC administration improves whole-body insulin sensitivity in streptozotocin/high-fat diet-induced diabetes rodents. It was reported that MSC therapy increased GIR but there was not a complete restoration to that of a chow fed animal [61]. We achieved comparable results employing insulin clamps to show the MSC-treated animals display greater systemic insulin sensitivity. Mice receiving MSC displayed an approximately 40% increase in the GIR compared to SHAM mice. Although the MSCs were administered via an intramyocardial injection, previous reports have identified MSC to persist, albeit low in magnitude, in peripheral tissues such as the lungs, spleen and liver [64,65]. As such, a systemic effect was not completely unanticipated. Moreover, the current study provides further support for a system-wide elevation in insulin-mediated glucose uptake by combining the insulin clamp with isotopic techniques to evaluate tissue-specific glucose utilization. In addition to increased cardiac glucose uptake, the soleus, superficial vastus lateralis, gastrocnemius and white adipose tissue exhibited an elevated rate of insulin-stimulated glucose uptake following MSC transplantation. The rise in glucose utilization in a range of insulin-sensitive tissues suggests the stem cell therapy is indeed acting in a systemic fashion rather than solely via tissue-specific mechanisms.

To address the mode by which the MSC treatment promoted glucose uptake in the selected tissues, GLUT4 protein was examined. Both gastrocnemius and white adipose tissue total GLUT4 protein was elevated in the MSC-treated mice. In the current study plasma fasting and experimental insulin concentrations were comparable between groups, however, there was an insulin-dependent influence conferred by the MSC transplantation. The gastrocnemius and white adipose tissue displayed higher p-Akt and an elevated p-Akt-to-Akt ratio. This suggests the cell therapy also augments the PI3K-Akt pathway to enhance glucose uptake.

The comprehensive *in vivo* approach employed for evaluating insulin sensitivity, substrate uptake and cardiac function following diet-induced insulin resistance and a standardized MI in the conscious mouse provides a greater understanding of the influence of MSC therapy on the diabetic infarcted heart. Despite this, a few readily apparent limitations exist. First, it is not clear if the improved ejection fraction is the result of direct cardio-protection or systemic alterations. The improved insulin sensitivity may give the heart added metabolic flexibility to meet its energetic requirements. Similarly, the reduction in plasma NEFAs may lessen OXPHOS uncoupling for more efficient ATP synthesis. In experimental models, ad libitum feeding of mice a high-fat diet with 55–60% of the caloric content being derived from fatty acids induces cardiovascular irregularities. After 16 weeks of HFF, systolic and diastolic blood pressure is markedly elevated compared to chow fed mice (11% calories by fat) [66]. Park et al. [67] identified C57BL/6 mice to exhibit impaired systolic performance following 20 weeks of HFF as indicated by fractional shortening. The animals evaluated in the current study were HFF for 13 weeks. As such, the therapeutic importance of peripheral glucose uptake and reduced hyperglycemia may increase as the dietary intervention is prolonged.

Another potential limitation of the present study is that the animals were not weight-matched at the time of glucose uptake evaluation. In the infarct-only animals, body weight was significantly lower following the MI event. This was associated with a reduced accumulation of body fat. Many of the metabolic aberrations associated with the HFF model are connected to the induction of obesity (fat deposition) [68]. Thus, the increase in the metabolic index of glucose uptake in the adipose tissue of the MI + PBS group is believed to be the result of the MI insult limiting comparable weight gain in these mice.

Finally, given the metabolic parameters were performed in the peri-infarct region of the heart, differential cell viability may be of modest concern. In the clinical setting, tissue salvage and infarct size reduction is based on the assumption that the core of the infarct is severely ischemic and necrotic [69]. Moreover, this core is surrounded by an area of jeopardized tissue called the peri-infarct region that may be salvaged by reperfusion efforts [69]. In experimental ligation models this is not observed. Rather than a progressive transition from infarct to viable tissue there is an abrupt, but irregular, changeover from ischemic zone to perfused, viable tissue [69,70]. While this is a limitation in matching the clinical setting exactly, it is also an advantage to the studies conducted because it largely minimizes the influence of apoptotic cells in the peri-infarct region following the MI event.

## Conclusions

In summary, the novel findings of this study are the MSC-mediated increase in both cardiac glucose uptake and mitochondrial OXPHOS efficiency. These metabolic improvements may assist in greater ATP provision for contractile demands following a MI. Our results also demonstrate MSC transplantation to reduce hyperglycemia, hyperlipidemia and improve peripheral tissue insulin-mediated glucose uptake. These findings differ from the effects of MSC therapy in the absence of type 2 diabetes and/or its metabolic abnormalities. Previous work by the authors evaluating the therapeutic influence of MSC transplantation on cardiac glucose utilization post-MI in chow fed mice did not identify MSC-induced alterations in cardiac glucose metabolism [18]. Furthermore, no apparent whole body effect on insulin sensitivity was mediated by MSC transplantation [18]. By utilizing a combined diet-induced insulin resistant/MI model the current study identifies that not only does MSC transplantation potentially lessen impairment in cardiac contractile performance via augmentation of cardiac-specific metabolism but also holds the ability to reach farther than the heart to dampen systemic dysregulation that may contribute to the maladaptive alterations in cardiac function.

## Abbreviations

2-[14C]DG: 2-[^14^C]deoxyglucose; CI: Mitochondrial electron transport chain complex 1; CII: Mitochondrial electron transport chain complex 2; CIII: Mitochondrial electron transport chain complex 3; CIV: Mitochondrial electron transport chain complex 4; CV: Mitochondrial electron transport chain complex 5; ATP: Synthase; CSA: Citrate synthase activity; GAPDH: glyceraldehyde-3-phosphate dehydrogenase; GIR: Glucose infusion rate; HFF: High-fat fed; HKII: Hexokinase 2; LAD: Left anterior descending coronary artery; LV: Remote left ventricle; MI: Myocardial infarction; MSC: Mesenchymal stem cell; OXPHOS: Oxidative phosphorylation; PCr: Phosphocreatine; PGC-1α: Peroxisome proliferator-activated receptor gamma coactivator-1alpha; PI: Peri-infarct region of left ventricle; RCR: Respiratory control ratio; Rg: Metabolic index of glucose uptake; UCP: Uncoupling protein; V0: State 2 respiration supported by complex I substrates malate, glutamate and pyruvate; VMAX-CI: State 3 respiration supported by complex I substrates malate, glutamate and pyruvate; VMAX-CI+CII: State 3 respiration via convergent electron input through complex I and II.

## Competing interests

The authors declare that they have no competing interests.

## Authors’ contributions

CCH, DHW, JNR and JS did the conception and design of the experiments. CCH, LM, FDJ, DBP and ZW performed the experiments and collected data. CCH and JS analyzed the data. CCH drafted the manuscript. All authors reviewed, edited and approved the final version of this manuscript.
